# The effect of academic self-efficacy on academic achievement among university students: a moderated mediation model of achievement goal orientation and teacher’s transformational leadership

**DOI:** 10.3389/fpsyg.2026.1674113

**Published:** 2026-02-19

**Authors:** Chang Liu, Xiaona Liu, Hu Gao

**Affiliations:** 1 Foreign Languages Department, Hengshui University, Hengshui, China; 2 Teacher Education Department, Hengshui University, Hengshui, China; 3 General Education Department, Shaanxi Vocational Academy of Arts, Xi'an, Shaanxi, China

**Keywords:** Social Cognitive Theory, academic self-efficacy, academic achievement, achievement goal orientation, transformational leadership

## Abstract

**Introduction:**

With the growing employment pressure faced by university students and the increasing demand for their comprehensive abilities, exploring the factors that influence academic achievement to promote their academic progress holds significant practical importance.

**Methods:**

Drawing from a sample of university students and grounded in the framework of Social Cognitive Theory, this study investigates the mechanism through which academic self-efficacy affects academic achievement. It specifically tests the mediating effect of achievement goal orientation and the moderating effect of teacher’s transformational leadership within this process.

**Results:**

The results indicate that: academic self-efficacy has a significant positive impact on the academic achievement of university students; achievement goal orientation plays a positive mediating role in the relationship between academic self-efficacy and academic achievement; and students’ perceived teacher’s transformational leadership positively moderates the relationship between academic self-efficacy and achievement goal orientation.

**Conclusion:**

Specifically, the positive effect of academic self-efficacy on achievement goal orientation is stronger when students perceive a higher level of transformational leadership from their teachers. This research confirms that academic self-efficacy drives academic achievement through a goal-setting pathway. The findings suggest that higher education institutions can promote student academic development by enhancing the transformational leadership capabilities of teachers, which in turn boosts students’ efficacy.

## 1 Introduction

Higher education plays a crucial role in building a workforce for the country’s rapid future development. Academic achievement reflects the effectiveness of talent cultivation in higher education and serves as a core indicator for measuring the quality of higher education (Tao et al., 2022). It is also a key factor in predicting students’ competitiveness in postgraduate entrance examinations and the job market (Al-Abyadh and Abdel Azeem, 2022). As a centralized manifestation of students’ holistic development, its formation is influenced by the interaction of individual, others, and environmental factors (Qureshi et al., 2023). Among these, academic self-efficacy, as a core personal factor, has become a critical pathway to enhancing academic achievement by driving learning motivation and strategy selection (Zhong et al., 2023). According to Social Cognitive Theory (Bandura, 2012), an individual’s behavior is determined by the dynamic interaction between personal factors and the environment. Research has confirmed that academic self-efficacy can not only directly predict academic achievement but also exert its influence indirectly through variables such as classroom engagement (Jiang et al., 2023; Meng and Zhang, 2023).

Goal-Setting Theory further suggests that individuals with high self-efficacy are more inclined to set challenging goals and optimize their strategies (Saks, 2024). Moreover, achievement goal orientation has a significant positive effect on academic achievement (Alasqah, 2022). It is plausible that academic self-efficacy influences academic achievement through the mediating role of achievement goal orientation. However, this mechanism may be constrained by environmental factors. According to the Conservation of Resources Theory (Hobfoll, 1988), leaders serve as a valuable situational resource for employees, influencing their goal commitment through supportive behaviors. Transformational leadership, as a supportive leadership style, enhances members’ understanding and identification with organizational goals by demonstrating charisma and articulating the organization’s vision and mission in an inspiring manner (Manu, 2022). In educational contexts, when students perceive their teachers exhibiting transformational leadership behaviors, they are more likely to increase their commitment to learning goals. Teachers who provide students with opportunities to express their ideas and offer personalized care and support can not only boost students’ learning motivation but also enhance their self-efficacy and guide their goal orientation (Saad Alessa, 2021). Thus, this study argues that teacher transformational leadership can be viewed as a critical environmental resource, where behaviors such as motivation, care, and intellectual stimulation may moderate the relationship between students’ self-efficacy and goal orientation. Accordingly, this paper integrates teacher transformational leadership into the research framework to examine its moderating role in this relationship.

Previous studies have largely focused on the direct impact of single variables on academic achievement, often overlooking the comprehensive effects of personal and environmental factors (Lee and Shute, 2010). Therefore, this study, grounded in the framework of Social Cognitive Theory, employs a moderated mediation model to simultaneously explore the influence of personal factors (academic self-efficacy and achievement goal orientation) and an environmental factor (teacher’s transformational leadership) on the academic achievement of university students. The research aims to examine how and when academic self-efficacy influences academic achievement through the mediating role of achievement goal orientation, and to investigate the moderating role of teacher’s transformational leadership in this process. This endeavor will not only help to uncover the mechanism for enhancing academic achievement through the interaction of personal and environmental factors but also provide evidence-based strategic recommendations for educators in higher education. This can better promote students’ academic development and address the pressing issue in current educational practice concerning how to effectively unlock student potential and optimize the learning environment.

## 2 Research hypotheses

### 2.1 Academic self-efficacy and university students’ academic achievement

Academic self-efficacy is a significant factor influencing academic achievement (Bhati et al., 2022; Figueiredo et al., 2024) and is closely linked to non-intellectual factors such as learning motivation, learning strategies, and academic emotions (Dong et al., 2023; Luo et al., 2023). Academic self-efficacy is the specific manifestation of self-efficacy in the academic domain. According to Bandura’s (1986) Social Cognitive Theory, this belief in one’s efficacy can manipulate an individual’s perspectives and actions, thereby affecting behavioral outcomes. Specifically, academic self-efficacy can be divided into two dimensions: learning ability self-efficacy and learning behavior self-efficacy (Meng and Zhang, 2023). Students with high learning ability self-efficacy tend to be more confident, dare to face academic challenges, and are willing to attempt new tasks. Even when faced with failure, they can maintain an optimistic attitude. This positive belief encourages them to continuously engage in learning and self-improvement, leading to excellent academic achievement (Zhao et al., 2024).

On the other hand, students with high learning behavior self-efficacy believe they can formulate and execute reasonable study plans, adopt effective learning strategies, and proactively seek solutions to problems. This positive behavioral attitude and choice help them overcome learning obstacles and achieve their academic goals, thereby enhancing their academic achievement (Figueiredo et al., 2024; Luo et al., 2023). In summary, learning ability self-efficacy and learning behavior self-efficacy are interconnected, and they jointly influence the academic achievement of university students. Students with high academic self-efficacy (in both ability and behavioral aspects) are more likely to exhibit positive learning attitudes and behaviors, adopt effective learning strategies, and maintain resilience when facing challenges, thus achieving better academic outcomes. Therefore, this study proposes the following hypothesis:

*H1*: Academic self-efficacy positively predicts the academic achievement of university students.

### 2.2 The mediating role of achievement goal orientation

Achievement goal orientation is a widely studied motivational variable in educational contexts (Miller et al., 2021), referring to an individual’s attitudinal tendency when selecting achievement goals, and it serves as a crucial source of motivation for learners to reach their goals (Li et al., 2021). Specifically, achievement goal orientation comprises two dimensions: learning goal orientation and performance goal orientation (Xu et al., 2000). Goal-setting theory posits that an individual’s motivation and performance are shaped by the clarity and difficulty of their goals (Saks, 2024). In academic contexts, academic self-efficacy—a student’s confidence in their ability to accomplish specific learning tasks—enhances goal commitment and drives the adoption of more challenging and well-defined learning objectives (Zhao et al., 2024). Students with high self-efficacy typically pursue learning-oriented goals, emphasizing skill growth and mastery, as they trust that effort yields progress. Conversely, those with low self-efficacy are more inclined toward performance-oriented goals (Luo et al., 2023). Hence, academic self-efficacy acts as a pivotal cognitive precursor to achievement goal orientation by governing how students select and pursue their goals.

Furthermore, studies indicate that students base their personal expectations on academic self-efficacy, which in turn directly or indirectly shapes their goal preferences and subsequent goal orientation (Saks, 2024). Liu et al. (2023) indicate that students with high academic self-efficacy tend to choose challenging goals, focus more on task engagement, and strive to achieve their objectives, resulting in a higher achievement goal orientation. Based on theoretical and empirical research, we infer that the higher a student’s academic self-efficacy, the greater their internal drive to complete learning tasks, the higher the learning goals they set, and consequently, the higher their achievement goal orientation.

Goal-setting theory further suggests that clear and challenging goals effectively boost an individual’s motivation and task performance (Saks, 2024). In educational settings, achievement goal orientation—referring to an individual’s cognitive perspective on the purpose of learning activities—directly impacts the quality of their goal setting and the consistency of their effort investment (Li et al., 2021). A learning goal orientation encourages students to treat learning as a process of skill development, leading them to set intrinsic goals focused on knowledge mastery and skill enhancement. This, in turn, increases their perseverance and strategic approach when encountering challenges, positively contributing to academic achievement (Xu et al., 2000). Conversely, a performance goal orientation might drive students to achieve objectives by surpassing peers or attaining high grades, which can also foster academic success in specific contexts (Xu et al., 2000). Thus, per goal-setting theory, distinct achievement goal orientations differentially influence academic outcomes by affecting the specificity and difficulty of goals set, as well as the sustained engagement in tasks. Empirical research has established achievement goal orientation as a predictor of academic achievement (Honicke et al., 2020; Miller et al., 2021). Enhancing this orientation helps students better manage study time and maintain focus on learning objectives, thereby improving academic performance. It also promotes active learning to accomplish tasks, which ultimately enhances academic achievement (Scherrer et al., 2020). In conclusion, the academic self-efficacy of university students may enhance their achievement goal orientation, which in turn influences their academic achievement. Therefore, we propose the following hypothesis:

*H2*: Achievement goal orientation mediates the relationship between academic self-efficacy and academic achievement.

### 2.3 The moderating role of teacher’s transformational leadership

According to Bandura (1977)'s Social Learning Theory, people can learn by observing the behavior of others. Transformational leadership refers to a process where leaders elevate their followers’ level of awareness regarding the importance and value of specific and idealized goals, encouraging them to prioritize the interests of the group or organization and to pursue higher-order needs (Manu, 2022). Many studies generally concur that perceiving a higher level of transformational leadership can positively influence an individual’s achievement goal orientation (Hannah et al., 2020; Layaman et al., 2021).

Although academic self-efficacy affects achievement goal orientation, this effect may be influenced by teacher’s transformational leadership. According to the Conservation of Resources (COR) theory, individuals experience significant stress and reduce their goal commitment when they perceive a lack of resources; conversely, they increase their goal commitment when resources are abundant. Both personal and environmental resources affect goal commitment, and an interaction exists between them (Radford, 2024). For example, the study by Li and Tsai (2020) found that the internal organizational environment moderated the impact of employees’ commitment to learning on their goal orientation; when employees perceived a positive internal environment, the effect of their learning commitment on goal orientation was stronger. Similarly, teacher’s transformational leadership, as an environmental resource, may moderate the effect of self-efficacy, a personal resource, on goal orientation. Furthermore, in their research on transformational leadership, Wu and Lee (2020) found that to become a transformational leader, one must possess sufficient resources. When leaders have ample resources, it can create a “spiral of resource gain,” making it easier to achieve goals and acquire new resources.

University students’ learning unfolds within the process of teacher-student interaction. While self-efficacy is undoubtedly important, teacher support also has a significant impact on students’ goal setting. When teacher’s transformational leadership is high, students perceive that their goal completion has strong external support. In this situation, the promoting effect of self-efficacy on goal setting may be enhanced, with the teacher’s transformational leadership acting as a catalyst. Conversely, when transformational leadership is low and external support for goal achievement is lacking, the effect of self-efficacy on goal setting may be diminished. Therefore, we propose the following hypothesis:

*H3*: Teacher's transformational leadership moderates the relationship between academic self-efficacy and achievement goal orientation. Specifically, the effect of academic self-efficacy on achievement goal orientation is stronger for university students who perceive a higher level of teacher's transformational leadership.

**Figure 1:**
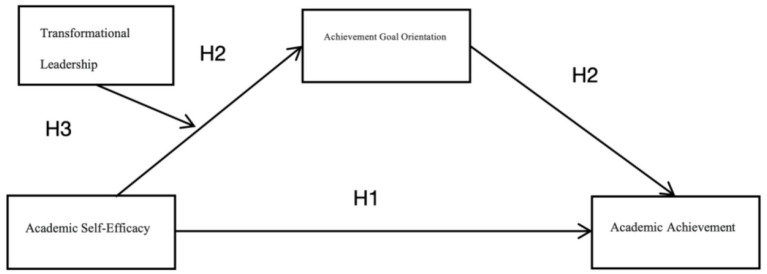
The research framework.

Based on the analysis above, this study establishes a research framework as shown in Figure 1.

## 3 Methodology

### 3.1 Sample and procedure

Data for this study were collected using a dedicated survey administered to university students from three institutions in Hebei Province, China. The choice of this student population was informed by several considerations. Hebei is a major province in higher education, where universities place strong emphasis on systematically fostering students’ academic achievement, resulting in regionally distinctive educational practices. However, under the coordinated development framework of Beijing–Tianjin–Hebei, higher education resources in Hebei objectively fall behind those in Beijing and Tianjin. Consequently, university students in Hebei often exhibit relatively lower levels of competence and academic outcomes, which in turn expose them to more intense competition and structural challenges in the job market. In this context, examining the pathways to improving the academic achievements of students in Hebei’s universities holds not only significant theoretical value but also pressing practical importance. To this end, the present study draws on samples from three representative universities in Hebei, ensuring strong alignment between the research subjects and the study objectives. This provides the empirical foundation for systematically testing the interrelationships among academic self-efficacy, achievement goal orientation, and teacher transformational leadership, and for building a theoretical model tailored to improving academic achievement in regional contexts.

We first contacted the deans of academic affairs at the selected universities, explaining that our research focused on academic achievement among college students. With institutional approval, questionnaires were distributed and data collected through online platforms. Participants were informed about the study’s purpose, anonymity, and voluntary participation, and small gifts were offered to encourage higher response rates. This study selected university students from three higher education institutions in Hebei Province as its research subjects, distributing questionnaires and collecting data via an online platform. A combination of purposive and convenience sampling was employed. First, purposive sampling was used to select one university from each of three different disciplinary categories in Hebei Province: a normal university, a science and engineering university, and a comprehensive university. The students from these three institutions are known to have a relatively high level of academic achievement among universities in Hebei Province. Second, convenience sampling was used to administer the questionnaire to students within these three universities.

According to the sampling standard proposed by Ghiselli et al. (1981), the minimum sample size should be at least 10 times the total number of items. As the four scales used in this study comprise a total of 69 items, a minimum of 690 valid questionnaires was required. A total of 792 questionnaires were distributed across the three universities. Invalid questionnaires were excluded based on the following criteria: completion time exceeding 20 min; completion time less than 5 min; and obvious signs of random responses. After removing 42 invalid questionnaires, a total of 750 valid questionnaires were collected. Participants included 360 males (48%) and 390 females (52%); there were 246 first-year students (32.8%), 227 s-year students (30.3%), 193 third-year students (25.7%), and 84 fourth-year students (11.2%).

### 3.2 Measures

#### 3.2.1 Academic self-efficacy

The Academic Self-Efficacy Scale developed by Liang (2004) was used to measure the level of academic self-efficacy. The scale consists of two dimensions and a total of 22 items. Students rated their academic self-efficacy on a 5-point Likert scale, from 1 (*Strongly disagree*) to 5 (*Strongly agree*). A higher score indicates a higher level of academic self-efficacy. In the present study, the Cronbach’s *α* coefficient was 0.954.

#### 3.2.2 Achievement goal orientation

The goal orientation scale revised by Xu et al. (2000) from Button et al.'s (1996) questionnaire was used to measure achievement goal orientation. It includes 12 items across two dimensions: performance goal orientation and mastery goal orientation. Students rated their achievement goal orientation on a 5-point Likert scale, from 1 (*Strongly disagree*) to 5 (*Strongly agree*). A higher score indicates a higher level of achievement goal orientation. In the present study, the Cronbach’s *α* coefficient was 0.872.

#### 3.2.3 Teacher’s transformational leadership

The Transformational Leadership Scale developed by Bass and Avolio (2000) was adopted, measuring four dimensions: idealized influence, inspirational motivation, intellectual stimulation, and individualized consideration, with 16 items. The items were modified for an educational context, changing “leader” to “teacher” and “subordinate” to “student.” The scoring was revised to a frequency scale from 1 (*Never*) to 5 (*Always*) to verify students’ perception of leadership. A higher score indicates a stronger perception of the teacher’s transformational leadership. In the present study, the Cronbach’s α coefficient was 0.914.

#### 3.2.4 Academic achievement

The scale developed by Li et al. (2016) was selected to measure students’ academic achievement. It comprises four dimensions: learning cognitive ability, communication ability, self-management ability, and interpersonal promotion ability, with a total of 19 items. Each item was rated on a 5-point Likert scale, with higher scores indicating higher academic achievement. In the present study, the Cronbach’s α coefficient was 0.925.

### 3.3 Data analysis

After standardizing all variables, the moderated mediation model was tested using the SPSS PROCESS Macro (Hayes, 2017). The bootstrapping method, based on 5,000 random samples, was used to generate 95% confidence intervals. We first tested the mediation effect of achievement goal orientation using Model 4, and then tested the moderated mediation effect of teacher’s transformational leadership using Model 7.

## 4 Results

### 4.1 Test for common method bias

As the same respondents rated all variables in the questionnaire, common method bias was a potential concern. To assess this, Harman’s single-factor test was conducted using exploratory factor analysis in SPSS 24.0 (Aguirre-Urreta and Hu, 2019). All survey items were included in an unrotated factor analysis. The criterion for the absence of severe common method bias is that the first factor should account for less than 50% of the total variance. The Kaiser-Meyer-Olkin (KMO) measure was 0.955, exceeding the recommended threshold of 0.800. Bartlett’s test of sphericity was significant (χ^2^ = [value would typically be reported here], *p* < 0.001), indicating the data were suitable for factor analysis. The analysis extracted 12 factors with eigenvalues greater than 1. The first factor explained 28.495% of the variance, which is below the 50% threshold. Therefore, common method variance is not a significant issue in this dataset, and further data analysis was warranted.

### 4.2 Descriptive statistics and correlation analysis

Table 1 presents the descriptive statistics and correlation analysis for all variables. The findings indicate that academic self-efficacy was significantly and positively correlated with achievement goal orientation (*r* = 0.49, *p* < 0.001), teacher’s transformational leadership (*r* = 0.41, *p* < 0.001), and academic achievement (*r* = 0.56, *p* < 0.001). Achievement goal orientation was significantly and positively correlated with teacher’s transformational leadership (*r* = 0.27, *p* < 0.001) and academic achievement (*r* = 0.54, *p* < 0.001). Teacher’s transformational leadership was also significantly and positively correlated with academic achievement (*r* = 0.42, *p* < 0.001). The correlation coefficients suggest no serious multicollinearity issues.

**Table 1:** Descriptive statistics and correlations (*N* = 750).

Variables	M	SD	1	2	3	4
1. Academic self-efficacy	3.46	0.687	–			
2. Achievement goal orientation	2.74	0.807	0.492**	–		
3. Teacher’s transformational leadership	3.71	0.591	0.409**	0.266**	–	
4. Academic achievement	3.54	0.602	0.564**	0.537**	0.421**	–

**p < 0.001.

### 4.3 Test of the mediating effect of achievement goal orientation

**Figure 2:**
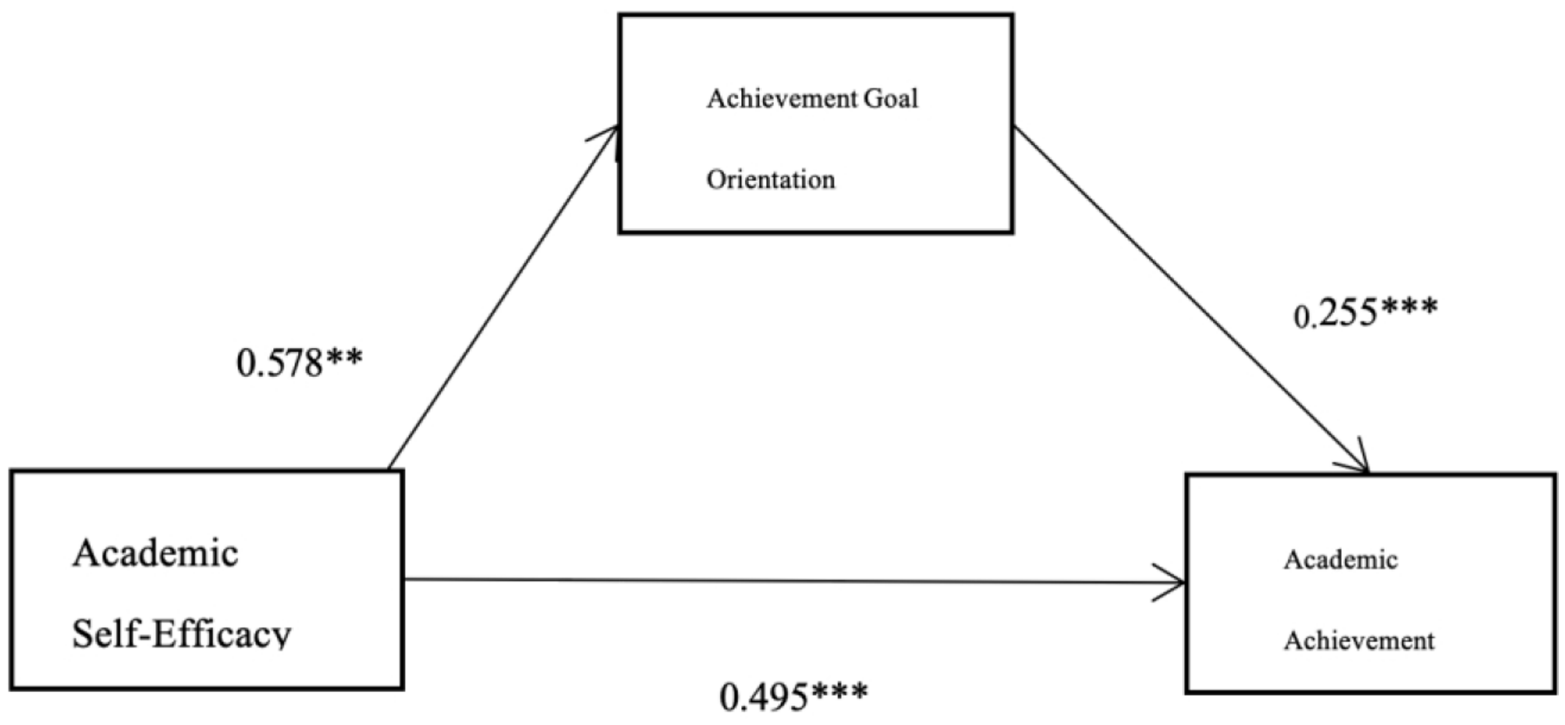
Mediation model diagram. **p < 0.01; ***p < 0.001.

Model 4 of the PROCESS Macro was used to test the mediating effect. As shown in Table 2, academic self-efficacy significantly and positively predicted academic achievement (*β* = 0.50, *p* < 0.001), supporting H1. Academic self-efficacy also positively predicted achievement goal orientation (*β* = 0.58, *p* < 0.001). When achievement goal orientation was included as a mediator, both academic self-efficacy (*β* = 0.35, *p* < 0.001) and achievement goal orientation (*β* = 0.26, *p* < 0.001) significantly predicted academic achievement. The indirect effect of achievement goal orientation was significant (*β* = 0.15, 95% CI [0.118, 0.179]), accounting for 29.7% of the total effect. This indicates that achievement goal orientation partially mediates the relationship between academic self-efficacy and academic achievement, supporting H2 (Figure 2).

**Table 2:** Results of the mediation analysis.

Predictor	Outcome: AGO	Outcome: AA	Outcome: AA
	Model 1	Model 2	Model 3
	β (SE)	β (SE)	β (SE)
Constant	0.740 (0.132)*	1.826 (0.093)*	1.637 (0.089)*
AS	0.578 (0.037)*	0.495 (0.026)*	0.347 (0.028)*
AGO			0.255 (0.024)*
*R* ^2^	0.242	0.319	0.407
*F*	238.771*	349.747*	256.598*
Indirect Effect	Effect	Boot SE	LLCI
AS → AGO → AA	0.147	0.016	0.118

AS = Academic Self-Efficacy; AGO = Achievement Goal Orientation; AA = Academic Achievement. All variables were standardized. LLCI = Lower Limit Confidence Interval; ULCI = Upper Limit Confidence Interval. *p* < 0.001. *p < 0.05.

### 4.4 Test of the moderating effect of teacher’s transformational leadership

Model 7 of the PROCESS Macro was used to test the moderating role. As shown in Table 3, the interaction term between academic self-efficacy and teacher’s transformational leadership significantly predicted achievement goal orientation (*β* = 0.41, *p* < 0.001). The index of moderated mediation was significant (Index = 0.105, 95% CI [0.078, 0.135]), confirming the moderated mediation model.

**Table 3:** Results of the moderated mediation model.

Predictor	Outcome: AGO (Model 1)	Outcome: AA (Model 2)
	β (SE)	β (SE)
Constant	2.673 (0.027)**	2.840 (0.068)**
AS	0.525 (0.040)**	0.347 (0.028)**
TL	0.125 (0.046)*	
AS × TL	0.413 (0.060)**	
AGO		0.255 (0.024)**
*R* ^2^	0.292	0.407
*F*	102.403**	256.598**

Bootstrap sample = 5,000. AS = Academic Self-Efficacy; AGO = Achievement Goal Orientation; TL = Teacher’s Transformational Leadership; AA = Academic Achievement. **p < 0.01, *p < 0.001.

**Figure 3:**
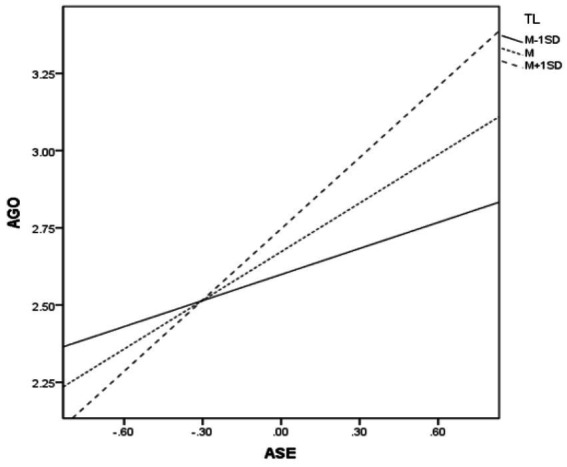
Transformational leadership moderates the relationship between academic self-efficacy and achievement goal orientation.

To interpret the effect, a simple slope analysis was conducted (see Table 4). The results indicate that the positive effect of academic self-efficacy on achievement goal orientation was stronger for students who perceived a high level of transformational leadership (Effect = 0.20, *p* < 0.001) compared to those who perceived a low level (Effect = 0.07, *p* < 0.001). This indicates that higher perceived teacher’s transformational leadership enhances the positive effect of academic self-efficacy on achievement goal orientation, supporting H3 (Figure 3).

**Table 4:** Conditional indirect effect of academic self-efficacy on academic achievement via achievement goal orientation.

Moderator: teacher’s transformational leadership	TL level	Effect	Boot SE	LLCI	ULCI
	Low (M − 1SD)	0.072	0.014	0.045	0.100
	Mean	0.134	0.015	0.106	0.163
	High (M + 1SD)	0.196	0.020	0.158	0.235

## 5 Discussion

This study investigated the mechanisms linking academic self-efficacy to academic achievement among university students, finding that academic self-efficacy positively influences academic achievement, with achievement goal orientation acting as a mediator and teacher’s transformational leadership moderating the first stage of this mediation.

### 5.1 Academic self-efficacy and academic achievement

The finding that academic self-efficacy significantly predicts academic achievement aligns with previous research (Bhati et al., 2022; Figueiredo et al., 2024). University students with high academic self-efficacy hold positive expectations, which transforms into intrinsic motivation. By integrating resources and optimizing strategies, these students convert their efficacy beliefs into superior academic performance (Zhao et al., 2024).

### 5.2 The mediating role of achievement goal orientation

This study reveals that achievement goal orientation partially mediates the relationship between academic self-efficacy and academic achievement. This contributes a “motivation-driven” perspective from Achievement Goal Theory (Elliot and Church, 1997) to a literature that often focuses on a “strategy-driven” view. Students with high self-efficacy who adopt a mastery goal orientation are more likely to choose challenging tasks, use deep learning strategies, and show resilience (Alhadabi and Karpinski, 2020; Senko and Tropiano, 2016), which ultimately enhances academic achievement. This finding provides new empirical evidence, expanding on prior research that identified mediators like learning strategies and academic emotions (Guo and Leung, 2021; Lüftenegger et al., 2016).

### 5.3 The moderating role of teacher’s transformational leadership

A key finding is that teacher’s transformational leadership positively moderates the link between academic self-efficacy and achievement goal orientation. This aligns with the Conservation of Resources (COR) theory (Hobfoll, 1988), where environmental resources (transformational leadership) strengthen the effect of individual resources (self-efficacy). This is consistent with findings in organizational behavior where leadership enhances the effects of psychological capital (Baig et al., 2021; Wang et al., 2014). By applying this to a higher education context, this study expands the traditional boundaries of leadership theory. When teachers exhibit transformational behaviors, students’ self-efficacy is more effectively converted into a clear achievement goal orientation. This finding clarifies the interaction pathway between individual and environmental resources in an educational setting and reveals the boundary conditions under which self-efficacy influences goal orientation.

Simultaneously, the findings demonstrate that in environments with low levels of transformational leadership, even high self-efficacy is less effectively channeled into a learning goal orientation. This suggests that self-efficacy does not automatically foster adaptive goal orientations; rather, the process through which motivation is directed is highly contingent upon the quality of contextual support. This insight challenges the conventional social cognitive tenet that “self-efficacy directly drives behavior,” highlighting the critical role of situational factors in motivation formation.

Moreover, this study is the first in higher education to identify the differential moderating role of transformational leadership. It robustly strengthens the positive effect of academic self-efficacy on learning goal orientation, while its influence on performance goal orientation is comparatively weaker. This indicates that transformational leadership, by emphasizing mastery and personal growth, steers students to apply their self-beliefs toward knowledge acquisition and self-improvement rather than a narrow focus on grades.

Diverging from prior research which has primarily examined its direct or mediating effects on academic achievement (Figueiredo et al., 2024; Guo and Leung, 2021), this study innovatively conceptualizes it as a moderator within the “individual-environment” resource interaction. It is confirmed to function not merely as external support but also as a catalyst for “resource gain,” enhancing the efficiency with which personal psychological assets are converted into productive goals. This finding not only broadens the application of Conservation of Resources Theory in educational research but also offers a novel lens for understanding how teacher leadership cultivates deep learning motivation.

## 6 Theoretical contributions and limitations

### 6.1 Theoretical contributions

Our study offers several key theoretical contributions to the literature. First, it extends Social Cognitive Theory by investigating the impact of academic self-efficacy on university students’ academic achievement. While existing research has established the beneficial effects of academic self-efficacy on achievement (Lei et al., 2022; Meng and Zhang, 2023), few empirical studies have examined the moderating role of teacher leadership in this relationship. Our findings elucidate the conditional effects of academic self-efficacy on achievement, thereby enriching the literature on this construct. This research underscores the positive association between academic self-efficacy and academic achievement, not only highlighting its significance but also identifying a novel antecedent.

Second, grounded in Social Cognitive Theory, this study proposes and tests a theoretical mechanism—mediated through achievement goal orientation—to explain how academic self-efficacy influences university students’ learning behaviors. Our results demonstrate that academic self-efficacy shapes the standards students set for themselves, motivates them to strive for personal breakthrough, and enhances their capacity to complete learning tasks more effectively. Prior studies on predictors of academic achievement have primarily focused on the independent effects of either academic self-efficacy or achievement goal orientation (Scherrer et al., 2020; Zhao et al., 2024), largely overlooking the potential mediating role of goal orientation. Consequently, our research emphasizes the critical importance of integrating these constructs within the Social Cognitive Framework to understand their interrelated effects.

Third, utilizing the lens of Conservation of Resources Theory, our study provides novel insights into the moderating effect of teacher transformational leadership. The results indicate that perceived transformational leadership strengthens the positive influence of academic self-efficacy on adaptive goal orientations; conversely, in its absence, this effect is attenuated. Therefore, this research not only deepens the understanding of how teacher leadership functions within the nexus of self-efficacy and goal setting but also offers valuable implications for how leadership styles can channel psychological resources toward productive academic outcomes.

### 6.2 Limitations

This study has several limitations that should be addressed in future research. First, although data were collected from multiple sources to mitigate common method bias concerns, the cross-sectional design inherently limits the ability to draw strong causal inferences regarding the hypothesized relationships. Future studies should employ longitudinal or experimental designs to better establish the causal effects of academic self-efficacy.

Second, as data were collected solely from Hebei Province, China, the generalizability of our findings to other regions, countries, or cultural contexts remains uncertain. Future research could seek to replicate these findings using more diverse samples from various geographical and cultural settings.

Finally, while our study focused on the moderating role of teacher transformational leadership, university students’ academic achievement is likely influenced by a multitude of other environmental factors. Future investigations could productively explore the impact of additional contextual variables, such as institutional climate or family environment, on academic achievement.

## Data Availability

The raw data supporting the conclusions of this article will be made available by the authors, without undue reservation.

## References

[ref1] Aguirre-Urreta M. I. Hu J. (2019). Detecting common method bias: performance of the harman’s single-factor test. ACM SIGMIS Database 50, 45–70. doi: 10.1145/3330472.3330477

[ref2] Al-Abyadh M. H. A. Abdel Azeem H. A. H. (2022). Academic achievement: influences of university students’ self-management and perceived self-efficacy. J. Intelligence 10:55. doi: 10.3390/jintelligence10030055, 35997411 PMC9396977

[ref3] Alasqah S. S. (2022). Goal orientation and its impact on university students’ academic achievement during the COVID-19 pandemic. SAGE Open 12:21582440221093617. doi: 10.1177/21582440221093617

[ref4] Alhadabi A. Karpinski A. C. (2020). Grit, self-efficacy, achievement orientation goals, and academic performance in university students. Int. J. Adolesc. Youth 25, 519–535. doi: 10.1080/02673843.2019.1679202

[ref5] Baig S. A. Iqbal S. Abrar M. Baig I. A. Amjad F. Zia-ur-Rehman M. . (2021). Impact of leadership styles on employees’ performance with moderating role of positive psychological capital. Total Qual. Manag. Bus. Excell. 32, 1085–1105. doi: 10.1080/14783363.2019.1665011

[ref6] Bandura A. (1977). Self-efficacy: toward a unifying theory of behavioral change. Psychol. Rev. 84, 191–215. doi: 10.1037/0033-295X.84.2.191, 847061

[ref7] Bandura A. (1986). Social foundations of thought and action: A social cognitive theory. Englewood Cliffs, NJ: Prentics-Hall.

[ref8] Bandura A. 2012 “Social cognitive theory,” Handbook of theories of social psychology: Volume 1 Lange P. A. M. Van Kruglanski A. W. Higgins E. T. London SAGE Publications 349–374

[ref9] Bass B. M. Avolio B. J. (2000). MLQ: Multifactor leadership questionnaire: Technical report, leader form, rater and scoring key for MLQ (form 5x-short). Redwood City, CA: Mind Garden.

[ref10] Bhati K. Baral R. Meher V. (2022). Academic self-efficacy and academic performance among undergraduate students in relation to gender and streams of education. Indones. J. Contemp. Educ. 4, 80–88. doi: 10.33122/ijoce.v4i2.35

[ref11] Button S. B. Mathieu J. E. Zajac D. M. (1996). Goal orientation in organizational research: a conceptual and empirical foundation. Organ. Behav. Hum. Decis. Process. 67, 26–48. doi: 10.1006/obhd.1996.0063

[ref12] Dong J. Y. Hassan N. C. Hassan A. B. Chen D. Guo W. (2023). Effect of achievement motivation and self-efficacy on general well-being among students at normal universities in Ningxia: the mediating role of time management. Behav. Sci. 14:15. doi: 10.3390/bs14010015, 38247667 PMC10813115

[ref13] Elliot A. J. Church M. A. (1997). A hierarchical model of approach and avoidance achievement motivation. J. Pers. Soc. Psychol. 72, 218–232. doi: 10.1037/0022-3514.72.1.218 10234849

[ref14] Figueiredo E. Fonseca C. Paiva T. (2024). Self-efficacy and academic performance in higher education: a case study. Eur. Public Soc. Innov. Rev. 9, 1–16. doi: 10.31637/epsir-2024-960

[ref15] Ghiselli E. E. Campbell J. P. Zedeck S. (1981). Measurement theory for the behavioral sciences. San Francisco, CA: W.H. Freeman and Company.

[ref16] Guo M. Leung F. K. S. (2021). Achievement goal orientations, learning strategies, and mathematics achievement: a comparison of Chinese Miao and Han students. Psychol. Schools 58, 107–123. doi: 10.1002/pits.22424

[ref17] Hannah S. T. Perez A. L. U. Lester P. B. Quick J. C. (2020). Bolstering workplace psychological well-being through transactional and transformational leadership. J. Leadersh. Organ. Stud. 27, 222–240. doi: 10.1177/1548051820933623

[ref18] Hayes A. F. (2017). Introduction to mediation, moderation, and conditional process analysis: A regression-based approach. New York: Guilford Publications.

[ref19] Hobfoll S. E. (1988). The ecology of stress. New York: Hemisphere Publishing Corporation.

[ref20] Honicke T. Broadbent J. Fuller-Tyszkiewicz M. (2020). Learner self-efficacy, goal orientation, and academic achievement: exploring mediating and moderating relationships. High. Educ. Res. Dev. 39, 689–703. doi: 10.1080/07294360.2019.1685941

[ref21] Jiang L. M. Zhang S. Li X. Luo F. (2023). How grit influences high school students’ academic performance and the mediation effect of academic self-efficacy and cognitive learning strategies. Curr. Psychol. 42, 94–103. doi: 10.1007/s12144-020-01306-x

[ref22] Layaman L. Harahap P. Djastuti I. Jaelani A. Djuwita D. (2021). The mediating effect of proactive knowledge sharing among transformational leadership, cohesion, and learning goal orientation on employee performance. Bus. Theory Pract. 22, 470–481. doi: 10.3846/btp.2021.13365

[ref23] Lee J. Shute V. J. (2010). Personal and social-contextual factors in K–12 academic performance: an integrative perspective on student learning. Educ. Psychol. 45, 185–202. doi: 10.1080/00461520.2010.493471

[ref24] Lei W. N. Wang X. W. Dai D. Y. Guo X. P. Xiang S. Q. Hu W. P. (2022). Academic self-efficacy and academic performance among high school students: a moderated mediation model of academic buoyancy and social support. Psychol. Schs. 59, 885–899. doi: 10.1002/pits.22653

[ref25] Li H. Y. Majumdar R. Chen M.-R. A. Ogata H. (2021). GOAL-oriented active learning (GOAL) system to promote reading engagement, self-directed learning behavior, and motivation in extensive reading. Comput. Educ. 171:104239. doi: 10.1016/j.compedu.2021.104239

[ref26] Li D.-C. Tsai C.-Y. (2020). Antecedents of employees’ goal orientation and the effects of goal orientation on e-learning outcomes: the roles of intra-organizational environment. Sustainability 12:4759. doi: 10.3390/su12114759

[ref27] Li X. Y. Yang N. Liu Z. Y. (2016). An empirical study on the factors of college students’ academic achievement: taking the local colleges and universities as an example. Educ. Res. 37, 78–86.

[ref28] Liang Y. S. (2004). Correlation between self-efficacy to school work and mental health of university students. Chin. J. Tissue Eng. Res. 8, 4962–4963. doi: 10.3321/j.issn:1673-8225.2004.24.014

[ref29] Liu Q. Du X. J. Lu H. Y. (2023). Teacher support and learning engagement of EFL learners: the mediating role of self-efficacy and achievement goal orientation. Curr. Psychol. 42, 2619–2635. doi: 10.1007/s12144-022-04043-5

[ref30] Lüftenegger M. Klug J. Harrer K. Langer M. Spiel C. Schober B. (2016). Students’ achievement goals, learning-related emotions and academic achievement. Front. Psychol. 7:603. doi: 10.3389/fpsyg.2016.00603, 27199836 PMC4852289

[ref31] Luo Q. Chen L. C. Yu D. F. Zhang K. (2023). The mediating role of learning engagement between self-efficacy and academic achievement among Chinese college students. Psychol. Res. Behav. Manag. 16, 1533–1543. doi: 10.2147/PRBM.S401145, 37143904 PMC10153452

[ref32] Manu A. (2022). “Transformational leadership” in The philosophy of disruption: From transition to transformational change (Bingley: Emerald Publishing Limited), 67–77.

[ref33] Meng Q. Zhang Q. (2023). The influence of academic self-efficacy on university students’ academic performance: the mediating effect of academic engagement. Sustainability 15:5767. doi: 10.3390/su15075767

[ref34] Miller A. L. Fassett K. T. Palmer D. L. (2021). Achievement goal orientation: a predictor of student engagement in higher education. Motiv. Emot. 45, 327–344. doi: 10.1007/s11031-021-09881-7

[ref35] Qureshi M. A. Khaskheli A. Qureshi J. A. Raza S. A. Yousufi S. Q. (2023). Factors affecting students’ learning performance through collaborative learning and engagement. Interact. Learn. Environ. 31, 2371–2391. doi: 10.1080/10494820.2021.1884886

[ref36] Radford K. (2024). “Conservation of resources theory” in A guide to key theories for human resource management research. eds. Hutchings K. Michailova S. Wilkinson A. (Cheltenham: Edward Elgar Publishing), 59–66.

[ref37] Saad Alessa G. (2021). The dimensions of transformational leadership and its organizational effects in public universities in Saudi Arabia: a systematic review. Front. Psychol. 12:682092. doi: 10.3389/fpsyg.2021.682092, 34867578 PMC8635992

[ref38] Saks K. (2024). The effect of self-efficacy and self-set grade goals on academic outcomes. Front. Psychol. 15:1324007. doi: 10.3389/fpsyg.2024.1324007, 38605828 PMC11007134

[ref39] Scherrer V. Preckel F. Schmidt I. Elliot A. J. (2020). Development of achievement goals and their relation to academic interest and achievement in adolescence: a review of the literature and two longitudinal studies. Dev. Psychol. 56, 795–814. doi: 10.1037/dev0000898, 32052983

[ref40] Senko C. Tropiano K. L. (2016). Comparing three models of achievement goals: goal orientations, goal standards, and goal complexes. J. Educ. Psychol. 108, 1178–1192. doi: 10.1037/edu0000114

[ref41] Tao Y. Meng Y. Gao Z. Y. Yang X. D. (2022). Perceived teacher support, student engagement, and academic achievement: a meta-analysis. Educ. Psychol. 42, 401–420. doi: 10.1080/01443410.2022.2033168

[ref42] Wang H. Sui Y. Luthans F. Wang D. N. Wu Y. H. (2014). Impact of authentic leadership on performance: role of followers’ positive psychological capital and relational processes. J. Organ. Behav. 35, 5–21. doi: 10.1002/job.1850

[ref43] Wu W.-L. Lee Y.-C. (2020). Do work engagement and transformational leadership facilitate knowledge sharing? A perspective of conservation of resources theory. Int. J. Environ. Res. Public Health 17:2615. doi: 10.3390/ijerph17072615, 32290352 PMC7177304

[ref44] Xu F. Z. Zhu Z. X. Lin Z. (2000). Research on the measurement of goal orientation and it’s impact on academic performance. Psychol. Dev. Educ. 16, 1–6. doi: 10.3969/j.issn.1001-4918.2000.02.001

[ref45] Zhao Z. Q. Ren P. Yang Q. (2024). Student self-management, academic achievement: exploring the mediating role of self-efficacy and the moderating influence of gender insights from a survey conducted in 3 universities in America. Available online at: https://arxiv.org/abs/2404.11029 (Accessed October 23, 2025).

[ref46] Zhong J. J. Wen J. Li K. (2023). Do achievement goals differently orient students’ academic engagement through learning strategy and academic self-efficacy and vary by grade. Psychol. Res. Behav. Manag. 16, 4779–4797. doi: 10.2147/PRBM.S424593, 38035203 PMC10683660

