# Supplementary material for: The effect of academic self-efficacy on academic achievement among university students: a moderated mediation model of achievement goal orientation and teacher’s transformational leadership
Source: Front Psychol. 2026 Feb 19;17:1674113. doi: 10.3389/fpsyg.2026.1674113 (PMC12960127; doi:10.3389/fpsyg.2026.1674113)
Supplement: Supplementary file 1 [file Supplementary_file_1.docx]

The Effect of Academic Self-Efficacy on Academic Achievement among University Students: A Moderated Mediation Model of Achievement Goal Orientation and Teacher's Transformational Leadership

# Appendix A

**Transformational Leadership Scale**

*Instructions: The following statements relate to the transformational leadership aspects of your teacher. Please choose from the five options, from "Never" to "Always," based on your actual feelings.*

| Item | Never | Rarely | Sometimes | Often | Always |
| --- | --- | --- | --- | --- | --- |
| 1. The teacher makes students feel proud. |  |  |  |  |  |
| 2. The teacher is considerate of students. |  |  |  |  |  |
| 3. The teacher understands the future development direction of students. |  |  |  |  |  |
| 4. The teacher gives students a strong sense of direction and fills them with confidence. |  |  |  |  |  |
| 5. The teacher is optimistic and hopeful when discussing the future. |  |  |  |  |  |
| 6. The teacher enthusiastically discusses students' needs. |  |  |  |  |  |
| 7. The teacher can help students envision their academic and career prospects. |  |  |  |  |  |
| 8. The teacher expresses confidence in achieving goals. |  |  |  |  |  |
| 9. The teacher encourages students to rethink ideas they have never questioned before. |  |  |  |  |  |
| 10. The teacher encourages students to look at problems from different perspectives. |  |  |  |  |  |
| 11. The teacher encourages students to solve problems from different angles. |  |  |  |  |  |
| 12. The teacher proposes the latest and most effective learning methods. |  |  |  |  |  |
| 13. The teacher is willing to spend extra time teaching students. |  |  |  |  |  |
| 14. The teacher treats students like friends. |  |  |  |  |  |
| 15. The teacher considers students' different interests, needs, and abilities. |  |  |  |  |  |
| 16. The teacher helps students develop their personal strengths. |  |  |  |  |  |

# Appendix B

**Academic Self-Efficacy Scale**

*Instructions: The following statements relate to your academic self-efficacy. Please choose from the five options, from "Strongly disagree" to "Strongly agree," based on your actual feelings.*

| Item | Strongly disagree | Disagree | Uncertain | Agree | Strongly agree |
| --- | --- | --- | --- | --- | --- |
| 1. I believe I have the ability to achieve good grades in my studies. |  |  |  |  |  |
| 2. I believe I have the ability to solve problems I encounter in my studies. |  |  |  |  |  |
| 3. Compared to other students in my class, my learning ability is relatively strong. |  |  |  |  |  |
| 4. I believe I can grasp the content taught by the teacher in class in a timely manner. |  |  |  |  |  |
| 5. I believe I can apply what I have learned. |  |  |  |  |  |
| 6. Compared to other students in my class, my understanding of my major is broader. |  |  |  |  |  |
| 7. I like to choose challenging learning tasks. |  |  |  |  |  |
| 8. I believe I can understand the knowledge in textbooks and the content taught by the teacher well. |  |  |  |  |  |
| 9. I often choose learning tasks that are difficult but from which I can learn, even if it requires more effort. |  |  |  |  |  |
| 10. Even if my grade on an exam is very poor, I can calmly analyze the mistakes I made. |  |  |  |  |  |
| 11. Regardless of whether my grades are good or bad, I never doubt my learning ability. |  |  |  |  |  |
| 12. When studying, I always check whether I have mastered the content by asking and answering my own questions. |  |  |  |  |  |
| 13. When thinking about a problem, I can connect it with previously learned knowledge. |  |  |  |  |  |
| 14. I often find that while I am reading a book, I don't know what it means. |  |  |  |  |  |
| 15. When reading a book, I can connect the content with the knowledge I have already mastered. |  |  |  |  |  |
| 16. I find that I am always distracted in class and cannot listen attentively. |  |  |  |  |  |
| 17. I often cannot accurately summarize the main idea of the content I have read. |  |  |  |  |  |
| 18. I always highlight important parts in my textbooks or notebooks to help with my studies. |  |  |  |  |  |
| 19. When reviewing for an exam, I can integrate all the knowledge I have learned. |  |  |  |  |  |
| 20. When taking notes in class, I try to write down every word the teacher says, regardless of its significance. |  |  |  |  |  |
| 21. When doing homework, I always try to recall what the teacher said in class to do the assignment well. |  |  |  |  |  |
| 22. Even if the teacher does not require it, I will voluntarily do the exercises at the end of each chapter to test my mastery of the knowledge. |  |  |  |  |  |

# Appendix C

**Achievement Goal Orientation Scale**

*Instructions: The following statements relate to your achievement goal orientation. Please choose from the five options, from "Strongly disagree" to "Strongly agree," based on your actual feelings.*

| Item | Strongly disagree | Disagree | Uncertain | Agree | Strongly agree |
| --- | --- | --- | --- | --- | --- |
| 1. I would rather do things I am good at than things I am poor at. |  |  |  |  |  |
| 2. I feel very happy when I am doing something that I know I won't make mistakes on. |  |  |  |  |  |
| 3. My favorite things are those that I do best. |  |  |  |  |  |
| 4. I would feel smart if I did something without making any mistakes. |  |  |  |  |  |
| 5. I like to do things that I have done well in the past. |  |  |  |  |  |
| 6. I would feel smart if I did better than most other people on something. |  |  |  |  |  |
| 7. It is important for me to have the opportunity to do challenging work. |  |  |  |  |  |
| 8. If I fail to complete a difficult task, I will try harder the next time I do it. |  |  |  |  |  |
| 9. I am willing to do tasks from which I can learn new things. |  |  |  |  |  |
| 10. The opportunity to learn new things is important to me. |  |  |  |  |  |
| 11. I will try my best to improve myself based on past experiences. |  |  |  |  |  |
| 12. The opportunity to expand my abilities is important to me. |  |  |  |  |  |

# Appendix D

**Academic Achievement Scale**

*Instructions: The following statements relate to your learning outcomes. Please choose from the five options, from "Strongly disagree" to "Strongly agree," based on your actual feelings.*

| Item | Strongly disagree | Disagree | Uncertain | Agree | Strongly agree |
| --- | --- | --- | --- | --- | --- |
| 1. I can flexibly apply the knowledge I have learned. |  |  |  |  |  |
| 2. In class, I can easily understand what the teacher is saying. |  |  |  |  |  |
| 3. I can quickly grasp the key to solving a problem. |  |  |  |  |  |
| 4. I can always understand new knowledge and skills quickly. |  |  |  |  |  |
| 5. I can communicate clearly with others. |  |  |  |  |  |
| 6. I know how to change topics and can grasp the basic points of a conversation. |  |  |  |  |  |
| 7. I am a good listener and do not like to interrupt others. |  |  |  |  |  |
| 8. I am afraid of face-to-face communication with others. |  |  |  |  |  |
| 9. Unless someone speaks to me first, I am generally unwilling to start a conversation. |  |  |  |  |  |
| 10. I don't care about plans; I study only when I feel like it. |  |  |  |  |  |
| 11. I don't really have goals; I just let things happen naturally. |  |  |  |  |  |
| 12. I can manage my time reasonably. |  |  |  |  |  |
| 13. I often delay my plans due to laziness. |  |  |  |  |  |
| 14. I am very clear about the goals I want to achieve academically. |  |  |  |  |  |
| 15. I always take the initiative to help other students. |  |  |  |  |  |
| 16. I can care for and be considerate of other students well. |  |  |  |  |  |
| 17. I can control my behavior well in different situations. |  |  |  |  |  |
| 18. I can cooperate well with other students. |  |  |  |  |  |
| 19. I can get along well with others. |  |  |  |  |  |
